# Zinc Finger Protein ZBTB20 protects against cardiac remodelling post‐myocardial infarction via ROS‐TNFα/ASK1/JNK pathway regulation

**DOI:** 10.1111/jcmm.15961

**Published:** 2020-10-16

**Authors:** Fangfang Li, Yiming Yang, Chuanyou Xue, Mengtong Tan, Lu Xu, Jianbo Gao, Luhong Xu, Jing Zong, Wenhao Qian

**Affiliations:** ^1^ Department of Cardiology The Affiliated Hospital of Xuzhou Medical University Xuzhou China; ^2^ Institute of Cardiovascular Disease Research Xuzhou Medical University Xuzhou China

**Keywords:** apoptosis, ASK1/JNK1/2, cardiac remodelling, myocardial infarction, ZBTB20

## Abstract

This study aims to determine the efficacy of Zinc finger protein ZBTB20 in treatment of post‐infarction cardiac remodelling. For this purpose, left anterior descending (LAD) ligation was operated on mice to induce myocardial infarction (MI) with sham control group as contrast and adeno‐associated virus (AAV9) system was used to deliver ZBTB20 to mouse heart by myocardial injection with vehicle‐injected control group as contrast two weeks before MI surgery. Then four weeks after MI, vehicle‐treated mice with left ventricular (LV) remodelling underwent deterioration of cardiac function, with symptoms of hypertrophy, interstitial fibrosis, inflammation and apoptosis. The vehicle‐injected mice also showed increase of infarct size and decrease of survival rate. Meanwhile, the ZBTB20‐overexpressed mice displayed improvement after MI. Moreover, the anti‐apoptosis effect of ZBTB20 was further confirmed in H9c2 cells subjected to hypoxia in vitro. Further study suggested that ZBTB20 exerts cardioprotection by inhibiting tumour necrosis factor α/apoptosis signal‐regulating kinase 1 (ASK1)/c‐Jun N‐terminal kinase 1/2 (JNK1/2) signalling, which was confirmed by shRNA‐JNK adenoviruses transfection or a JNK activator in vitro as well as ASK1 overexpression in vivo. In summary, our data suggest that ZBTB20 could alleviate cardiac remodelling post‐MI. Thus, administration of ZBTB20 can be considered as a promising treatment strategy for heart failure post‐MI.

Significance Statement: ZBTB20 could alleviate cardiac remodelling post‐MI via inhibition of ASK1/JNK1/2 signalling.

## 1 INTRODUCTION

Myocardial infarction (MI) induced by coronary artery occlusion is the leading cause of morbidity and mortality worldwide.^1^ Cardiac remodelling always starts with acute myocardial infarction.^2^ Then hours to days after acute MI, ischaemia leads to myocardial necrosis, resulting in influx of inflammatory cells and subsequent activation of fibroblasts^2^, ^3^ which helps to clear dead cardiomyocytes and maintain ventricular shape.^2^, ^4^ However, in the long term, non‐infarct myocardium will undergo eccentric hypertrophy, cell death, further leading to left ventricular cavity dilation. The prevalence of post‐MI heart failure continues to rise as nowadays patients after myocardial infarction live longer compared to the past.^5^ However, current therapies to control cardiac remodelling process following MI are far from effective.^6^, ^7^ Therefore, it is essential to look for new treatment strategy to prevent adverse remodelling after MI.

c‐Jun N‐terminal kinase (JNK) is a crucial molecule that activates remodelling process after MI.^8^ As a pathway regulating various stress, activation of JNK is proved to induce cell apoptosis, inflammation, hypertrophy and fibrosis in cardiac remodelling post‐MI.^9^ JNK deficiency could reduce the risk of cardiac fibrosis and remodelling post‐MI.^10^ Thus, it could be considered as a promising therapeutic strategy for treatment of cardiac remodelling post‐MI to target JNK pathway‐mediated inflammation and cell death.

As a new member of the POK (POZ and Krüppel) family of transcriptional repressors, Zinc finger and BTB domain‐containing 20 (ZBTB20) contains an intact N‐terminal BTB domain and a C‐terminal zinc finger domain. It is reported that ZBTB20 participates in various cellular functions such as cellular proliferation, transcriptional regulation, ion channel assembly, tumorigenesis and chromatin remodelling. By regulating epithelial growth factor receptor expression, ZBTB20 promotes hepatocyte proliferation in mouse liver regeneration.^11^ Kan H et al found that by transcriptionally repressing FoxO1, ZBTB20 promotes HCC cell viability, proliferation, tumorigenicity and cell cycle progression.^12^ ZBTB20 also inhibits IκBα gene transcription, governs IκBα protein expression and then promotes Toll‐like receptor‐triggered innate immune responses by some reports.^13^ Versatility of ZBTB20 is suggested in all these studies. However, whether ZBTB20 can protect against cardiac remodelling after MI remains unclear. In our study, MI was performed on mice intentionally to induce cardiac remodelling for investigation of efficacy of ZBTB20 against cardiac remodelling.

## 2 MATERIALS AND METHODS

### 2.1 Reagents

The primary antibodies against the following proteins were purchased from Cell Signaling Technology (MA, USA): JNK1/2, phospho‐JNK1/2, p38, phospho‐p38, Bcl‐2, Bax, caspase 3, cleaved caspase 3 and GAPDH. The primary antibodies against ASK1 and phospho‐ASK1 were purchased from ZBTB20 Cruz Inc (TX, USA). The antibodies against tumour necrosis factor a (TNFa) were purchased from Abcam (Camb, Britain).

### 2.2 Animals

Institute of Laboratory Animal Science, Chinese Academy of Medical Sciences (Beijing, China) supplied adult male C57BL/6 mice (8‐10 weeks aged) to us. Mice were divided into four groups: AAV9‐vehicle‐sham group (n = 15), AAV9‐ZBTB20‐sham group (n = 15), AAV9‐vehicle‐MI group (n = 50), AAV9‐ZBTB20‐MI group (n = 50). Mice were subjected to AAV9‐ZBTB20 or AAV9‐vehicle injection two weeks before MI model to overexpress ZBTB20. In the mechanism exploring experiment, mice were divided into five groups: AAV9‐ASK1‐sham group (n = 15), AAV9‐ASK1+AAV9‐ZBTB20‐sham group (n = 15), AAV9‐ASK1‐MI group (n = 50), AAV9‐ASK1+AAV9‐ZBTB20‐MI group (n = 50) and AAV9‐vehicle‐MI group (n = 50). To overexpress ASK1, mice were injected with AAV9‐ASK1 two weeks before MI model. The Guide for the Care and Use of Laboratory Animals published by the U. S. National Institutes of Health and all animal procedures were applied in our experiment, and approval from the Institutional Animal Care and Use Committee at Xuzhou Medical University (Xuzhou, China) was also acquired.

### 2.3 Left coronary artery ligation surgery (LAD)

The left coronary artery ligation was conducted according to the previous guidance.^14^ In short, after anesthetization procedure, mouse chest was opened between the third and fourth intercostal spaces on the left side. After opening the pericardium, the proximal descending branch of the left coronary artery was ligated with 7~0 silk thread. The left coronary artery was not ligated in the sham group.

### 2.4 Recombinant Adeno‐Associated Virus (AAV) 9 construction

AAV9‐ASK1, AAV9‐ZBTB20 and AAV9‐vehicle were constructed and generated by Vigene Biosciences (Shandong, China). Briefly, ZBTB20 or ASK1 mice gene was cloned into p‐ENTER vector by AsiS I and Mlu I restriction sites. The p‐ENTER plasmid containing the desired gene and AAV vectors pAV‐C‐GFP was cotransfected into 293 cells to obtain pAAV‐MCS plasmid. Then, recombinant plasmid pAAV‐MCS was transfected into AAV‐293 cells. Three days after transfection, AAV9 vector‐producing 293T cells were harvested for vector purification. Real‐time PCR was used to quantify AAV viral particles.

### 2.5 Viral delivery protocol

Mice received heart injection of AAV9‐ZBTB20, AAV9‐ASK1 or AAV9‐vehicle (1 × 10^11^ viral particles) two weeks before surgery. In short, 3% pentobarbital sodium (80 mg/kg, intraperitoneal) was administered for anesthetization and a rodent ventilator was used to maintain artificial respiration. Left pleural cavity was opened between the left third and fourth ribs and then the pericardium. A 29‐gauge syringe was used for injection through the apical, anterior and lateral wall of the left ventricle (10 μL for each site). After the procedure, the chest was closed. Mice were injected with 0.5% bupivacaine for pain relief.

### 2.6 Echocardiographic and haemodynamic evaluation

Transthoracic echocardiography and haemodynamic analysis were used for detection as previous study described.^15^ 1.5% isoflurane was used to anaesthetize mice. Echocardiography was performed with a 10‐MHz linear‐array ultrasound transducer. A microtip catheter transducer was applied for haemodynamic measurements. Signals were recorded by a Millar Pressure‐Volume system.

### 2.7 Cardiac morphology and histomorphometric analysis

Cardiac morphology and histomorphometric analysis were performed as previous study described.^15^ Haematoxylin and eosin (H&E) staining was used to count cell surface area with more than 200 cells per group. Picrosirius Red (PSR) staining was used to count LV collagen volume with more than 10 fields for each heart. The infarct size was analysed by Image‐Pro Plus6.0 system (from the U. S. National Institutes of Health). Hearts of impermeable infarct were excluded from subsequent analysis to reduce the hybrid effect of biological end‐points caused by infarct volume variation. The cross‐sectional area (CSA) of the cells was analysed with quantitative digital image system based on (Image‐Pro Plus6.0).

Heart sections were also incubated with anti‐CD68 (ABclonal, A6554), anti‐CD45 (ABclonal, A2115) and anti‐TNFα (ab6671) for immunohistochemistry staining by DAB detection kit.

### 2.8 Measurements of MDA level and activity of GPx and SOD

The malondialdehyde (MDA), NADPH oxidase and the total superoxide dismutase (SOD) were detected as indicated by manufacturer (Biotein, Shanghai, China). ROS was detected by dichlorofluorescein diacetate assay (DCFH‐DA, Biotein, Shanghai, China). The cells were incubated with DCFH‐DA (10 μmol/L) for 60 minutes at 37°C, and immunofluorescence was detected using a fluorescence microplate reader (excitation wavelength/emission wavelength: 485/525 nm) or by light microscopy (BX51TRF; Olympus Corporation, Tokyo, Japan).

### 2.9 Detection of cell death

The commercial Tunel kit (Millipore, USA) was used to detect cell apoptosis in both cardiomyocytes and heart sections according to instructions from manufacturers. A fluorescence microscope (OLYMPUS DX51) was used to quantify in high times (400 x). A random examination of more than four surrounding areas of each slide was performed. Lactate dehydrogenase (LDH) levels was measured using commercial kit reagents (Biotein, Shanghai, China) according to instructions from manufacturer.

### 2.10 Quantitative polymerase chain reaction (qPCR)

Total RNA was extracted from either cardiac tissue of frozen mouse or cardiomyocytes using TRIzol (15596‐026; Invitrogen). RNA (2 μg of each sample) was reverse‐transcribed into cDNA using oligo (DT) primers and a Transcriptor First Strand cDNA Synthesis Kit (04896866001; Roche). PCR amplifications in all groups were quantified using a LightCycler 480 SYBR Green 1 Master Mix (04707516001; Roche), and the results were normalized against glyceraldehyde‐3‐phosphate dehydrogenase (GAPDH) gene expression. The promers used are listed in Table 1.

**Table 1:** Primer sequences used for qPCR

mRNA	Forward	Reverse
ANP	ACCTGCTAGACCACCTGGAG	CCTTGGCTGTTATCTTCGGTACCGG
BNP	GAGGTCACTCCTATCCTCTGG	GCCATTTCCTCCGACTTTTCTC
β‐MHC	CCGAGTCCCAGGTCAACAA	CTTCACGGGCACCCTTGGA
Col1agenI	AGGCTTCAGTGGTTTGGATG	CACCAACAGCACCATCGTTA
Col1agenIII	AAGGCTGCAAGATGGATGCT	GTGCTTACGTGGGACAGTCA
CTGF	AGGGCCTCTTCTGCGATTTC	CTTTGGAAGGACTCACCGCT
IL‐1β	CCGTGGACCTTCCAGGATGA	GGGAACGTCACACACCAGCA
IL‐6	AGTTGCCTTCTTGGGACTGA	TCCACGATTTCCCAGAGAAC
MCP1	TAAAAACCTGGATCGGAACCAAA	GCATTAGCTTCAGATTTACGGGT
TNFα	CATCTTCTCAAAATTCGAGTGACAA	TGGGAGTAGACAAGGTACAACCC
GAPDH	ACTCCACTCACGGCAAATTC	TCTCCATGGTGGTGAAGACA

Note

Sequences are listed 5′‐3′.

### 2.11 Western blotting

Ice‐cold radioimmunoprecipitation assay buffer (containing 50 mmol/L Tris‐Hcl, 150 mmol/L NaCl, 1% Triton X‐100, 1% sodium deoxycholate and 0.1% SDS) was used to extract protein from cardiomyocytes and heart tissue. Then protein was subjected to 10% SDS‐PAGE (50 μg per sample). After transferred onto immobilon membranes (Millipore, Billerica, MA, USA), proteins were incubated with primary antibodies overnight at 4°C. The primary antibodies included the following: ZBTB20 (#ab243143, Abcam, 1:1000 diluted), Bax (#2772), Bcl‐2 (#2870), c‐caspase3 (#9664), T‐caspase3 (#9661), TNFα (#11948), Phospho (P)‐ASK1 (Thr845), total (T)‐ASK1(Thr845)(# #3765), P‐JNK1/2 (#4668p), T‐JNK1/2(#9258), P‐ERK1/2 (#4370P), T‐ERK1/2 (#4695), P‐P38 (#4511P), T‐P38 (#9212P) and GAPDH (#2118, Cell Signaling Technology, 1:1000 diluted), And then incubated with second antibodies of either goat anti‐rabbit IgG (926‐32211; LI‐COR) or goat antimouse IgG (C11026‐03; LI‐COR) for one hour. Analysis and quantification was performed by an Odyssey infrared imaging system (LI‐COR Biosciences). The GAPDH was used as reference.

### 2.12 Elisa assay

Plasma levels of TNF‐α, IL‐6, MMP‐2 and MMP‐9 were quantified by using commercial ELISA kits (Technology Co., LTD, Boster, Biological, China).

### 2.13 Cardiomyocyte culture

H9c2 rat cardiomyocytes (Cell Bank of the Chinese Academy of Sciences, Shanghai, China) were grown in DMEM (C11995; Gibco, Grand Island, NY, USA) supplemented with 10% FBS (FBS, 10099; Gibco), penicillin (100 U/mL) and streptomycin (100 mg/mL) (15140; Gibco) in a humidified CO_2_ incubator (SANYO 18M, Osaka, Japan) with 5% CO_2_ at 37°C. A serum‐free DMEM was used to culture cells for 12 hours before stimulation. Cells were transfected with Ad‐ZBTB20 (MOI = 50) for 8 hours to overexpress ZBTB20.

Neonatal rat cardiomyocyte (NRCM) culture was performed as previous study described.^15^ Briefly, one‐ to two‐day‐old Sprague‐Dawley rats were killed by cervical dislocation. Hearts were quickly removed, and the ventricles were washed with PBS three times and incubated with 0.125% trypsin‐EDTA (Gibco, 2520‐072) for 15 minutes. Ventricles were then enzymatically digested four times for fifteen minutes each in 0.125% trypsin‐EDTA in PBS. Digestion was stopped by adding FBS at a final concentration of 10%. The cells were then centrifuged at 250 *g* for 8 minutes and resuspended in DMEM/F12 (Gibco, C11330) supplemented with 10% FBS. Resuspended cells were incubated for 1‐2 hours in a 100 mm dish to allow non‐cardiac myocytes (mainly cardiac fibroblasts) to adhere to the plastic. Cells were then placed in six well plates at a density of 5 × 10^5^ cells per well with 1% bromodeoxyuridine for 48 hours. Cells were transfected with Ad‐ZBTB20 (MOI = 50) or Ad‐vehicle for 8 hours to overexpress ZBTB20.^16^ Cells were treated with JNK activator anisomycin (40 ng/mL) to activate JNK1/2.^17^ The cell hypoxia model was induced as previous study described.^18^ Cells were incubated in a condition with 5% oxygen (O_2_) and 5% carbon dioxide (CO_2_) and 90% N_2_. Cells in control group were cultured in normal atmosphere with 5% CO_2_ and 95% air at 37℃.

To knock down JNK1/2, Ad‐shJNK adenoviruses (generated from Vigene biosciences. Shandong, China) were generated and infected into cells (MOI = 100).^16^ After infection and culture in a FBS‐free medium for 8 hours, cells were exposed to hypoxia for 24 hours.

### 2.14 Primary mouse heart endothelial cells and fibroblasts isolation

Mouse hearts were removed after four weeks of MI and washed in Hanks’ balanced salt solution buffer. Collagenase A was used to digest heart tissue. Cells were collected in 10% FBS‐DMEM‐F12 (Gibco). A nylon mesh (70‐mm pores) was used as filter. Endothelial cells (ECs) were harvested with CD31 beads in Hanks’ solution. After washing, ECs were seeded in dishes precoated with 2% gelatin (Sigma, Oakville, ON, Canada) and then cultured with 10% FBS‐DMEM‐F12 (Gibco).

For fibroblasts, mouse hearts were removed after four weeks of MI. Hearts were washed in Hanks’ balanced salt solution buffer. Collagenase A was used to digest the heart tissue. Cells were collected in 10% FBS‐DMEM‐F12 (Gibco). A nylon mesh (70‐mm pores) was used as filter. Cells were seeded in a 10 cm dish. After culturing for 90 minutes, fibroblasts stuck to the plate. Then, supernatant was removed. Fibroblasts were cultured with 10% FBS‐DMEM‐F12 (Gibco).

### 2.15 Statistical analysis

All data were expressed as mean ± SD. Differences among groups were handled by a two‐way analysis of variance followed by Tukey's post hoc test. Comparisons between two groups were analysed by an unpaired Student's t test. It was considered as statistically significant when *P* value was less than 0.05.

## 3 RESULTS

### 3.1 ZBTB20 improves outcome post‐MI

Expression level of ZBTB20 after MI was detested first. As shown in Figure S1, the expression level of ZBTB20 was down‐regulated in mouse heart after MI compared to the sham hearts and it was also down‐regulated in cardiomyocytes exposed to hypoxia (Figure S1A,B). ZBTB20 level in endothelial cells and fibroblasts in heart after MI was also detected. The expression level of ZBTB20 was changed neither in endothelial cells nor in fibroblasts in mouse heart after MI (Figure S1C,D).

**Figure 1:**
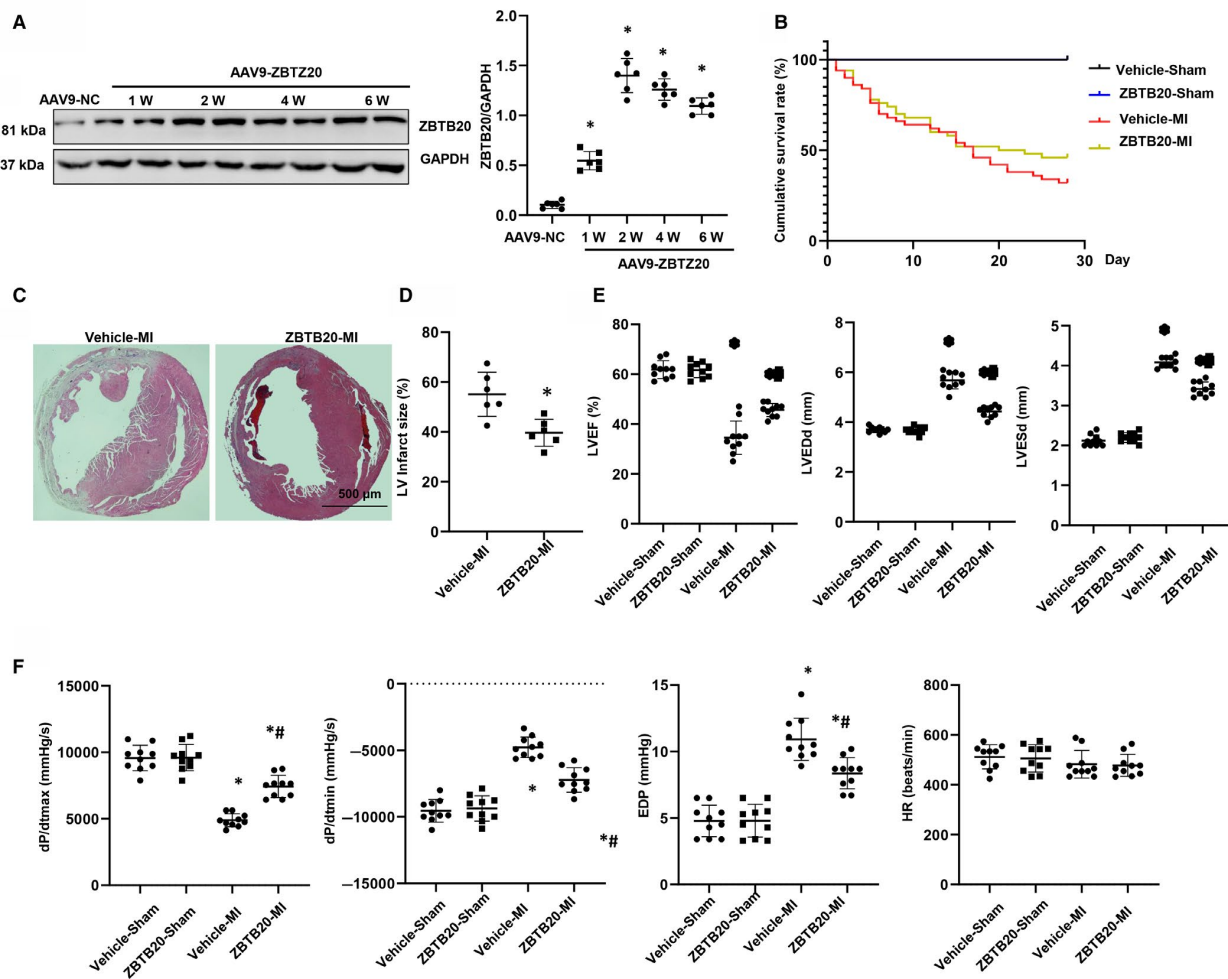
ZBTB20 ameliorates post‐MI outcomes. A, Expression of ZBTB20 in heart tissue 1, 2, 4 and 6 wk after AAV9‐ZBTO20 injection (n = 6). *P < 0.05 vs Con. B, Kaplan‐Meier survival analysis of mice in vehicle‐MI and ZBTB20‐MI groups in the first four weeks after MI (n = 100). C and D, H&E staining of mouse hearts in vehicle‐MI and ZBTB20‐MI groups four weeks after MI (n = 10, C, Representative image; D, Quantitative results). *P < 0.05 vs vehicle‐MI. E, Echocardiographic and haemodynamic (dP/dtmax, dP/dtmin, end‐diastolic pressure, heart rate) results for mice in the four groups at four weeks post‐MI (n = 10). *P < 0.05 vs vehicle‐sham; #*P* < 0.05 vs vehicle‐MI. One‐way analysis of variance followed by Tukey's post hoc test was used in Figure 1A. Two‐way analysis of variance followed by Tukey's post hoc test was used in Figure 1E,F. Unpaired Student's t test was used in Figure 1D

The expression of ZBTB20 in heart tissue increased after 1, 2, 4 and 6 weeks of AAV9‐ZBTB20 injection in mice (Figure 1A). Four weeks after MI, the survival rate in each group was calculated. As a result, a full period survival rate in the two sham groups was observed. However, the mortality rate was lower in ZBTB20‐overexpressed group than in the vehicle‐treated group (Figure 1B). H&E staining result showed that mice in the vehicle‐group had higher infarct size, while mice in the ZBTB20‐overexpressed group showed lower infarct size (Figure 1C,D). Furthermore, cardiac dysfunction was evaluated by echocardiography and haemodynamic measurements. After four weeks of MI, LVEF, dP/dtmax and dP/dtmin increased; LVEDd, LVESd and end‐diastolic pressure were reduced in ZBTB20‐overexpressed mice. The heart rate remained unchanged among four groups (Figure 1E,F).

### 3.2 ZBTB20 attenuates MI‐induced cardiac hypertrophy

**Figure 2:**
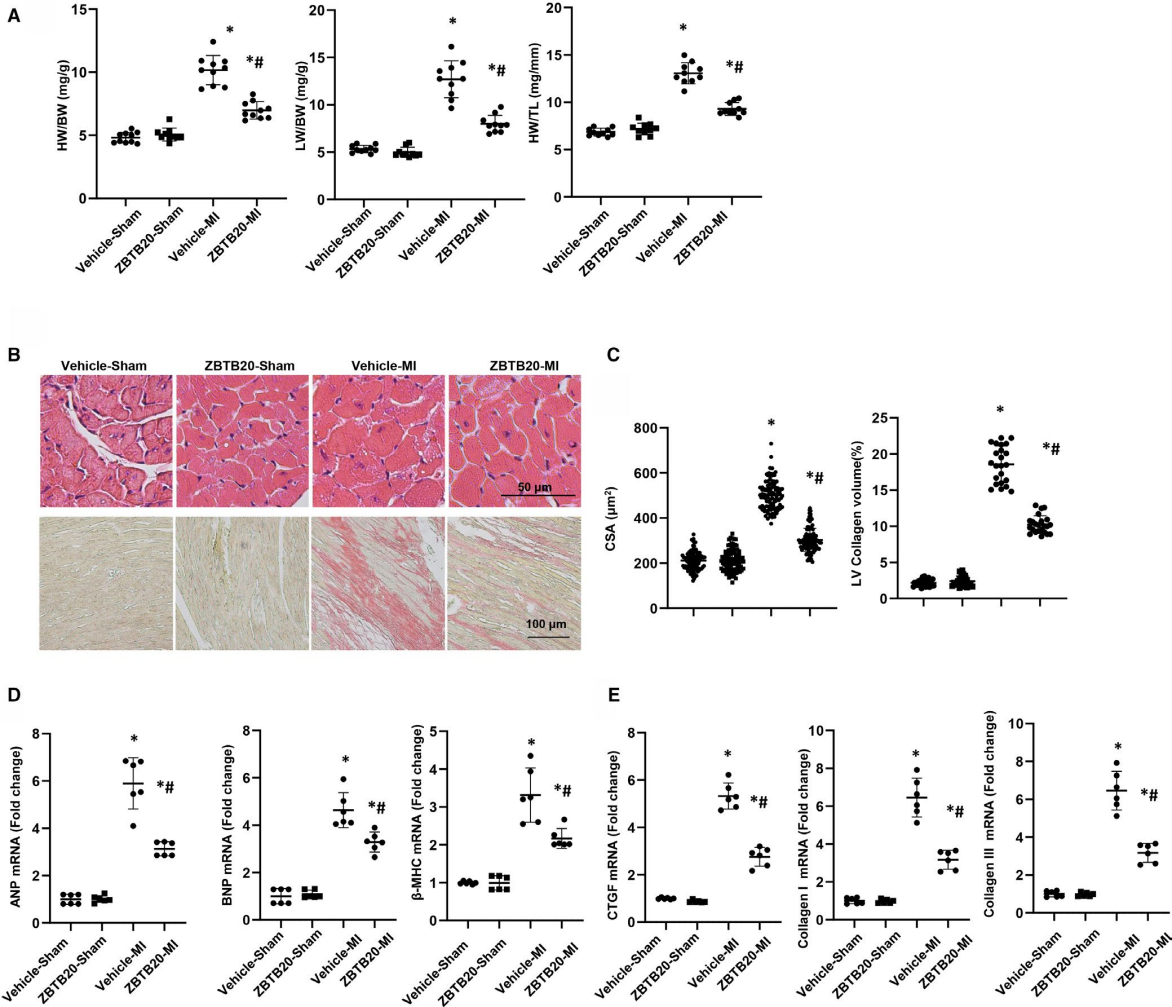
ZBTB20 attenuates MI‐induced cardiac hypertrophy. A, Statistical analysis of HW/BW, HW/TL and LW/BW ratios in mice from the vehicle and ZBTB20 groups (n = 20 per group). B, Sections were stained with H&E (n = 6; top) to analyse the cross‐sectional area (CSA, n ≥ 100 cells per experimental group). PSR staining of heart tissue (bottom) to analyse the LV collagen volume (n ≥ 25 fields per experimental group). C, Statistical analysis of the CSA and LV collagen volume (%). D and E, Relative mRNA levels of ANP, BNP, β‐MHC, CTGF, collagen I and collagen III in LV samples from vehicle‐ and ZBTB20‐overexpressed hearts (n = 6). *P < 0.05 vs vehicle‐sham; ^#^ *P* < .05 vs vehicle‐MI. Two‐way analysis of variance followed by Tukey's post hoc test was used

Pathological cardiac hypertrophy and fibrosis are associated with cardiac remodelling post‐MI. Therefore, Cardiac hypertrophy and fibrosis was detected. Four weeks after MI, vehicle‐treated mice exhibited increased ratios of heart weight (HW)/body weight (BW), lung weight (LW)/BW and HW/tibial length (TL) compared with the sham‐operated group. ZBTB20‐overexpressing decreased these ratios (HW/BW, LW/BW, HW/TL) when compared with vehicle‐treated mice after MI (Figure 2A). H&E staining revealed that cell surface area (CSA) of vehicle‐treated mouse heart increased. ZBTB20‐overexpressed mice heart had a reduced CSA (Figure 2B,C). Meanwhile, vehicle‐mice heart revealed increased interstitial fibrosis. ZBTB20‐overexpressed mice heart revealed lower interstitial fibrosis (Figure 2B,C). Additionally, the increased level of mRNA as hypertrophic and fibrotic marker was reduced in ZBTB20‐overexpressed mice four weeks post‐MI (Figure 2D,E).

### 3.3 ZBTB20 inhibits MI‐induced inflammatory responses and oxidative stress

**Figure 3:**
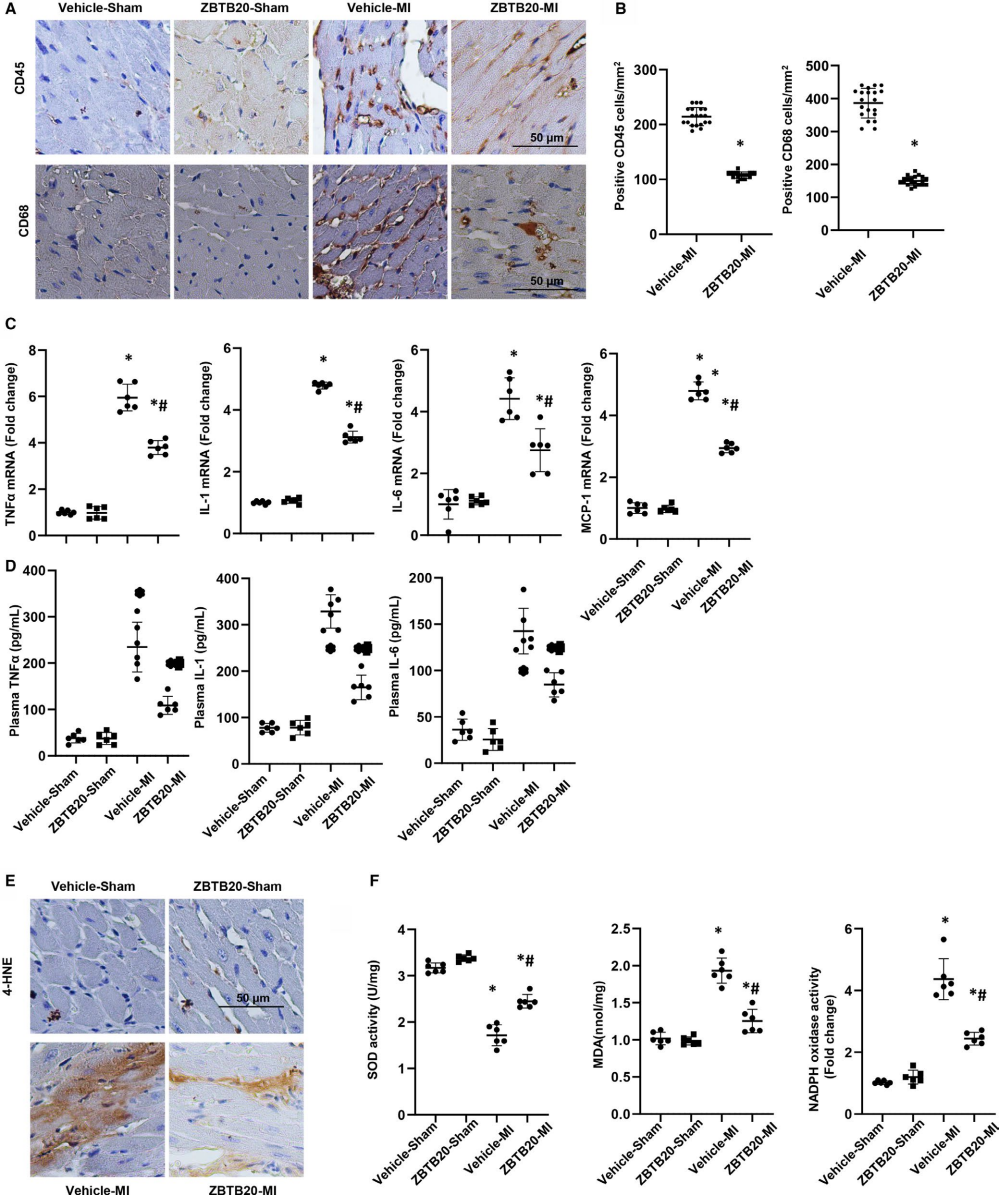
ZBTB20 inhibits MI‐induced inflammatory responses and oxidative stress. A, Immunohistochemical staining showing the number of CD45‐ and CD68‐positive cells in the heart cross‐sections (n = 6). B, Statistical analysis of the number of CD45‐ and CD68‐positive cells in the indicated group (n > 10 field per heart). C, Relative mRNA levels of TNFα, MCP‐1, IL‐1 and IL‐6 in hearts (n = 6). D, TNFα, IL‐1 and IL‐6 level in mice plasma (n = 6). E, Immunohistochemical staining showing 4‐HNE in hearts (n = 6). F, Total SOD activity, MDA level and NADPH oxidase activity in hearts (n = 6). *P < 0.05 vs vehicle‐sham; ^#^ *P* < 0.05 vs vehicle‐MI. Two‐way analysis of variance followed by Tukey's post hoc test was used in Figure 3C,D,F. Unpaired Student's t test was used in Figure 1B

Prolonged inflammation leads to pathological remodelling. Thus, whether ZBTB20 affected the inflammatory response after MI was examined. Immunohistochemical staining showed obvious infiltration of CD45‐positive leucocytes and CD68‐positive macrophages in hearts from vehicle‐mice. The increased inflammatory cell infiltration was markedly reduced in ZBTB20‐overexpressed mice hearts (Figure 3A,B). Additionally, increased mRNA expression of inflammatory cytokines in vehicle‐treated mouse heart was reduced in ZBTB20‐overexpressed mice heart (Figure 3C). ZBTB20‐overexpressing also reduced plasma inflammatory cytokines level in mice after MI (Figure 3D). The increased level of 4‐HNE in remodelled hearts was mitigated by ZBTB20‐overexpressing (Figure 3E). Furthermore, decreased SOD enzymatic activity in remodelled hearts was reversed by ZBTB20 overexpressing (Figure 3F), whereas the increased activities of MDA and NADPH oxidase in remodelled hearts were suppressed by ZBTB20 overexpressing (Figure 3F).

### 3.4 ZBTB20 inhibits apoptosis in vivo and in vitro

**Figure 4:**
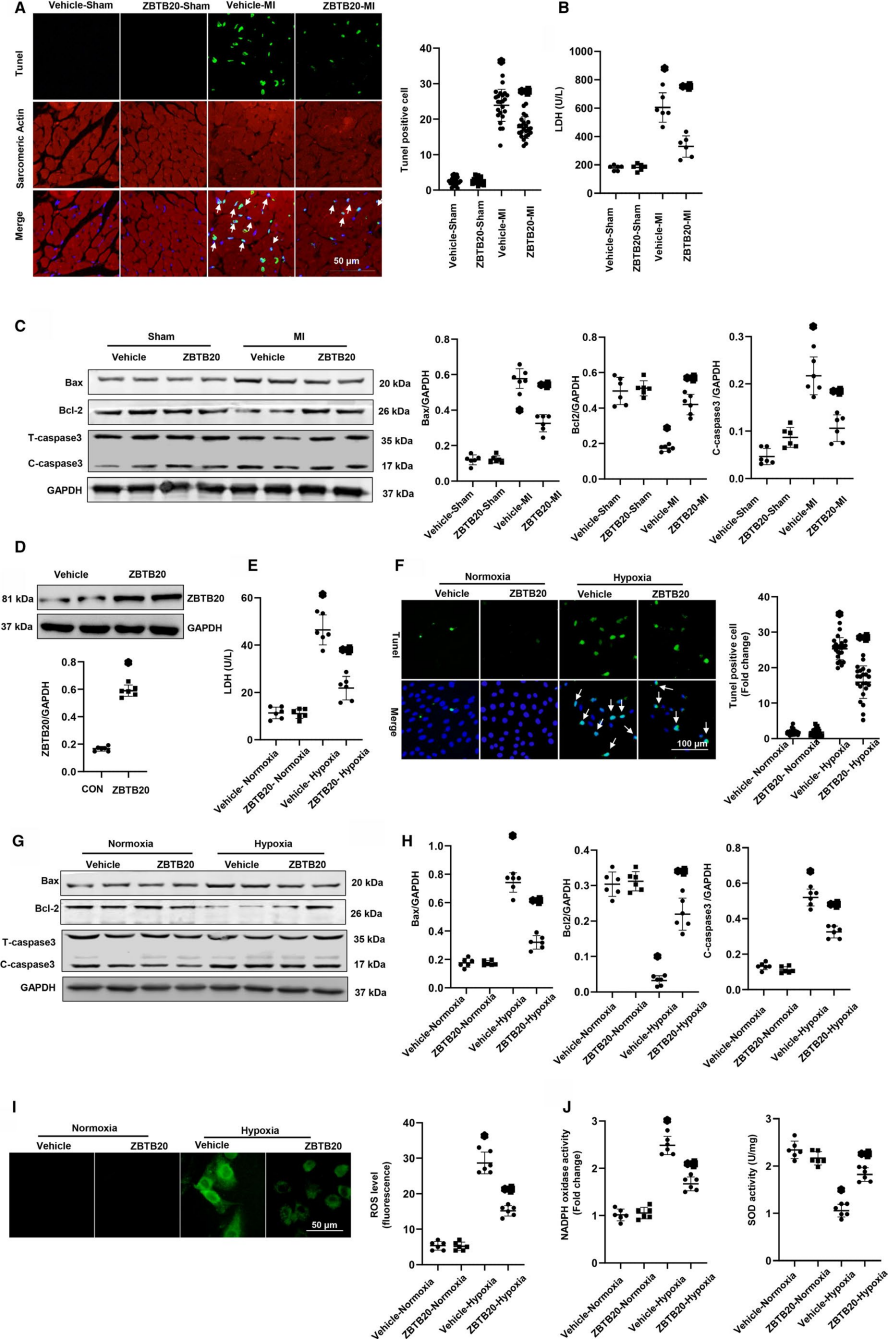
ZBTB20 inhibits apoptosis in vivo and in vitro. A, TUNEL staining (left) and quantitation (right) in the hearts at four weeks post‐MI (n = 6, *P < 0.05 vs vehicle‐MI). B, LDH level (n = 6). C, Representative western blots and quantitation of Bax, Bcl‐2 and C‐caspase 3 in the heart tissue (n = 6). *P < 0.05 vs vehicle‐sham; ^#^ *P* < 0.05 vs vehicle‐MI. D–H. H9c2 cells were transfected with Ad‐ZBTB20 for 8 h, then exposed to hypoxia for 24 h. D. The expression level of ZBTB20 after cell was transferred with Ad‐ZBTB20 (*P < 0.05 vs vehicle). E. LDH level (n = 6, *P < 0.05 vs hypoxia). F, TUNEL staining and quantitation in the indicated group (n = 6). G and H, Representative western blots (C) and quantitation (D) of Bax, Bcl‐2 and C‐caspase 3 in the indicated group (n = 6). I, ROS level in the cardiomyocytes (n = 6). J. NAPDH oxidase and SOD activity in the cardiomyocytes (n = 6). *P < 0.05 vs vehicle‐normoxia; ^#^ *P* < 0.05 vs vehicle‐hypoxia. Two‐way analysis of variance followed by Tukey's post hoc test was used in Figure 4A‐C,E,F,H‐J. Unpaired Student's t test was used in Figure 4D

As cardiomyocyte apoptosis is a main feature of remodelling post‐MI, it is necessary to determine the extent of apoptosis. A larger number of TUNEL‐positive cells in vehicle‐mice hearts and also a reduced TUNEL‐positive cell number in ZBTB20‐overexpressed hearts (Figure 4A) were observed. The LDH level in heart tissue was also reduced by ZBTB20 overexpression (Figure 4B). Western blot result showed that in vehicle‐mice, Bax and cleaved caspase 3 were elevated and Bcl‐2 was reduced (Figure 4B). In ZBTB20‐overexpressed mice, Bax and C‐caspase 3 were reduced and Bcl‐2 was elevated (Figure 4C).

Then, Ad‐ZBTB20 was used to overexpress ZBTB20 in H9c2 cells (Figure 4D) which were then subjected to hypoxia. Consequently, ZBTB20 increased cell viability which decreased after hypoxia stimulation. The percentage of TUNEL‐positive cells was reduced in ZBTB20‐overexpressed group (Figure 4F). The pro‐apoptotic markers (Bax and C‐caspase 3) dropped, and Bcl‐2 was elevated in ZBTB20‐overexpressed group compared with vehicle‐cells (Figure 4G,H). The anti‐oxidative stress effect of ZBTB20 in vitro was also determined. As shown in Figure 4I,J, ZBTB20 reduced the increased ROS level in cardiomyocytes exposed to hypoxia and also the activity of NADPH oxidase and increased the activity of SOD.

### 3.5 ZBTB20 inhibits the TNFα/ASK1/JNK1/2 pathway

**Figure 5:**
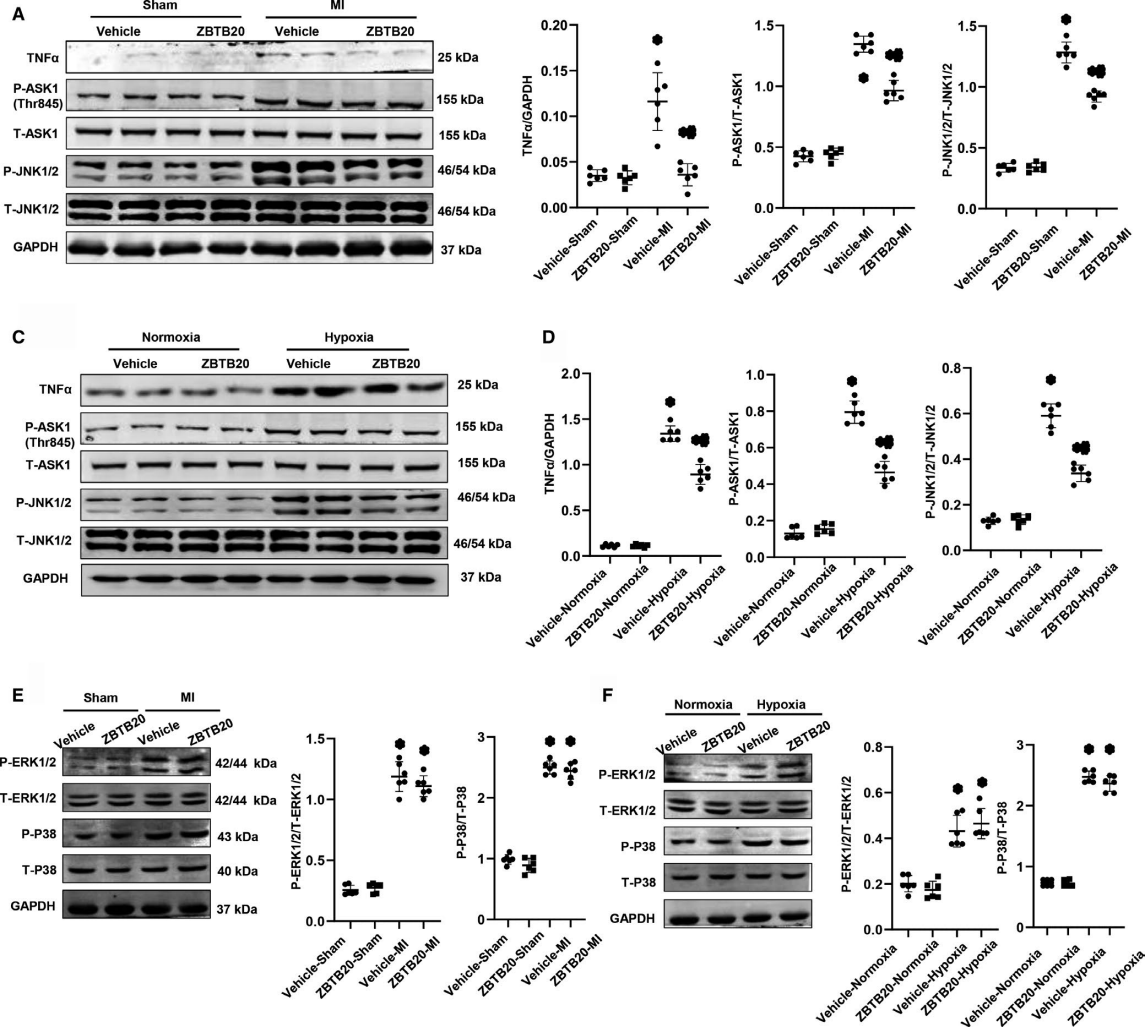
ZBTB20 inhibits MI‐induced activation of TNFα/ASK1/JNK1/2 pathway. A and B, Representative western blots (A) and quantitation (B) of TNFα, p‐ASK1, p‐JNK, total ASK1 and total JNK in heart tissue at four weeks after sham or MI surgery (n = 6, *P < 0.05 vs vehicle‐sham; ^#^ *P* < 0.05 vs vehicle‐MI). C and D, Representative western blots (C) and quantitation (D) of TNFα, p‐ASK1, p‐JNK, total ASK1 and total JNK in ZBTB20 overexpressed H9c2 cardiomyocytes after exposure to hypoxia for 24 h (n = 6). *P < 0.05 vs vehicle‐normoxia; ^#^ *P* < 0.05 vs vehicle‐hypoxia. E, Representative western blots and quantitation of p‐ERK1/2, p‐P38, total ERK1/2 and total P38 in heart tissue (n = 6, *P < 0.05 vs vehicle‐sham; ^#^ *P* < 0.05 vs vehicle‐MI). F, Representative western blots and quantitation of p‐ERK1/2, p‐P38, total ERK1/2 and total P38 in H9c2 cardiomyocytes (n = 6). *P < 0.05 vs vehicle‐normoxia; ^#^ *P* < 0.05 vs vehicle‐hypoxia. Two‐way analysis of variance followed by Tukey's post hoc test was used

Growing evidence demonstrates that the JNK1/2 signalling cascade is a determinant molecule driving cardiac remodelling. Therefore, the JNK pathway was investigated in this study. It was observed that mice in the vehicle‐MI group exhibited higher levels of TNFα expression and phosphorylation of ASK1(Thr845) and JNK1/2 (Figure 5A,B). By contrast, ZBTB20 overexpressing reduced the levels of TNFα and phosphorylation of ASK1 (Thr845) and JNK1/2 (Figure 5A,B). In cardiomyocytes subjected to hypoxia, ZBTB20 also decreased TNFα expression and phosphorylation of ASK1 (Thr845) and JNK1/2 (Figure 5C,D).

Other MAP3Ks molecules were also evaluated. As shown in Figure 5E,F, ERK1/2 and P38 activation level were increased in heart infarct tissue and cardiomyocytes exposed to hypoxia, but remained unchanged in ZBTB20‐treated heart tissue or cardiomyocytes with hypoxia.

### 3.6 ZBTB20 suppresses MI‐induced cardiac remodelling in a JNK1/2‐dependent manner

**Figure 6:**
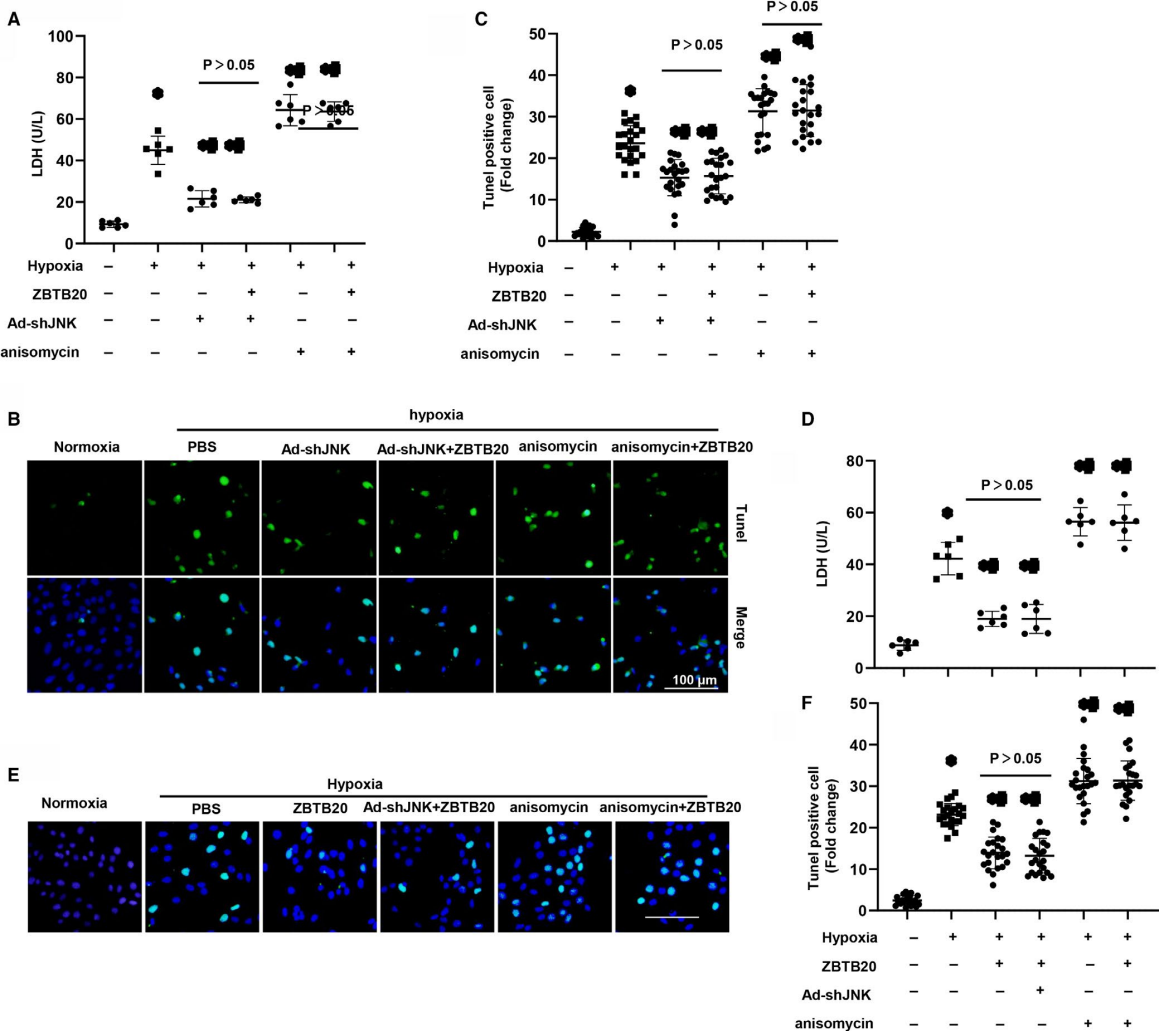
ZBTB20 suppresses MI‐induced cardiac remodelling in JNK1/2‐dependent manner. A–C, H9c2 cardiomyocytes were infected with Ad‐shJNK and/or Ad‐ZBTB20 or the JNK agonist anisomycin (40 ng/mL) for 8 h and exposed to hypoxia for 24 h. A, LDH level (n = 6). B and C, TUNEL staining (B) and quantitation (C) in the indicated group (n = 6). *P < 0.05 vs vehicle‐normoxia; ^#^ *P* < 0.05 vs vehicle‐hypoxia. Two‐way analysis of variance followed by Tukey's post hoc test was used. D–F, Neonatal rat cardiomyocytes were infected with Ad‐shJNK and/or Ad‐ZBTB20 or treated with the JNK agonist anisomycin (40 ng/mL) and exposed to hypoxia for 24 h. D, LDH level (n = 6). E and F, TUNEL staining (E) and quantitation (F) in the indicated group (n = 6). *P < 0.05 vs vehicle‐normoxia; ^#^ *P* < 0.05 vs vehicle‐hypoxia; ^ζ^ *P* < 0.05 vs ZBTB20‐hypoxia. Two‐way analysis of variance followed by Tukey's post hoc test was used

Cardiomyocytes were infected with Ad‐shJNK to knock down JNK1/2. Ad‐shJNK infection significantly reduced JNK signalling. Importantly, upon hypoxic stimulation, infection with Ad‐shJNK increased cell viability and decreased cell apoptosis. By contrast, under hypoxia condition, the apoptosis rate was unchanged between Ad‐shJNK group and ZBTB20 pretreatment + Ad‐shJNK group (Figure 6A–C). The JNK activator anisomycin (40 ng/mL) was also used in the present study.^17^ Notably, upon hypoxic stimulation, anisomycin abrogated the elevated cell viability and reduced cell apoptosis after ZBTB20 overexpressing (Figure 6A–C).

To evaluate the effects of ZBTB20 on neonatal rat cardiomyocytes (NRCMs), these cells were infected with Ad‐shJNK before pretreated with ZBTB20 and exposed to hypoxia for 24 hours. As expected, ZBTB20 increased cell viability and reduced cell apoptosis (Figure 6D–F). JNK silencing could not enhance protective effects of ZBTB20 and anisomycin completely abolished the effects of ZBTB20 on NRCMs (Figure 6D‐F).

### 3.7 ASK1 overexpression blocks the anti‐remodelling effects of ZBTB20 in vivo

**Figure 7:**
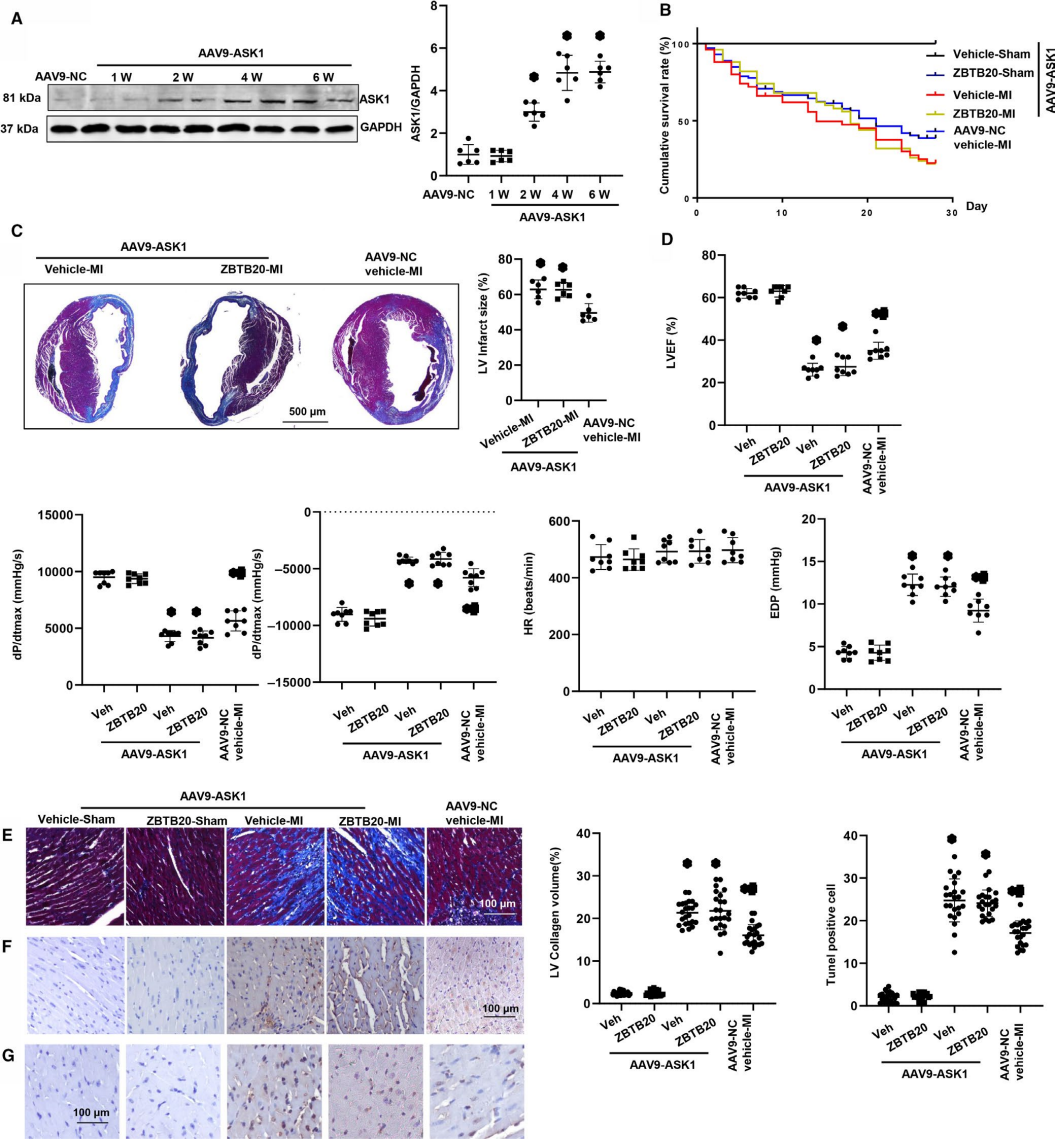
ASK1 overexpression blocks anti‐remodelling effects of ZBTB20 in vivo. Mice were subjected to myocardial injection of AAV9‐ASK1 and AAV9‐ZBTB20 or AAV9‐NC (NC: Negative Control) two weeks before LAD surgery. A, Expression of ASK1 1, 2, 4 and 6 wk after AAV9‐ASK1 injection (n = 6). *P < 0.05 vs Con. B, Kaplan‐Meier survival analysis of mice in vehicle‐MI and ZBTB20‐MI groups in the first four weeks after MI (n = 50). C, Masson staining of mice heart in vehicle‐MI and ZBTB20‐MI groups four weeks after MI (Left, representative image; Right, quantitation of infarction area). *P < 0.05 vs vehicle‐MI. D, Echocardiographic (LVEF) and haemodynamic (dP/dtmax, dP/dtmin, end‐diastolic pressure, heart rate) results for mice in the four groups at four weeks post‐MI (n = 6−8). E, Masson staining of heart tissue to analyse the LV collagen volume (n = 6 and >25 fields per experimental group; Left, representative image; Right, statistical analysis of the CSA and LV collagen volume [%]). F, Immunohistochemical staining showing TNFα release in the heart (n = 6). G, TUNEL staining (left) and quantitation (right) in the hearts (n = 6). *P < 0.05 vs vehicle‐sham. One‐way analysis of variance followed by Tukey's post hoc test was used in Figure 7A,C. Two‐way analysis of variance followed by Tukey's post hoc test was used in Figure 7D,E,G

To confirm the importance of ASK1/JNK signalling on ZBTB20‐mediated protection, AAV9‐ASK1 delivery system was used to overexpress ASK1 in mouse hearts. We found that ASK1 was up‐regulated in mouse heart tissues two weeks post‐injection and levels were persistently high for six weeks post‐injection (Figure 7A). ASK1 counteracted protective effects of ZBTB20 as demonstrated by the same survival rate (Figure 7B), infarction area (Figure 7C), cardiac dysfunction (Figure 7D), augmentation of cardiac fibrosis (Figure 7E), inflammation (Figure 7F) and apoptosis (Figure 7G). Cardiac remodelling was reduced in ASK1‐overexpressed infarcted mice compared with the control group (AAV9‐vehicle‐MI) as assessed by increased survival rate (Figure 7B), infarction area (Figure 7C), cardiac dysfunction (Figure 7D), augmented cardiac fibrosis (Figure 7E), inflammation (Figure 7F) and apoptosis (Figure 7G).

## 4 DISCUSSION

Revascularization and drug therapy in the early stages of MI significantly improve the survival rate after MI. But many patients suffer from heart failure caused by poor LV remodelling.^19^ Multiple pathophysiological factors play roles in remodelling the heart after MI and the fundamental determinants of this process include the extent of initial infarction and sufficiency of post‐MI repair, such as cardiomyocyte death, inflammatory response, fibrosis and compensatory hypertrophy of the non‐infarcted myocardium.^7^, ^20^ In the long term, the uncontrolled remodelling process may lead to irreversible effects on the heart.^21^ Therefore, new therapeutic strategies to treat the condition before heart failure are of great significance. Of note, new therapeutic agents that counteract apoptosis, inflammation, hypertrophy and interstitial fibrosis effects may be promising. This study demonstrates that ZBTB20 is a prospective candidate to prevent remodelling process post‐MI.

Various previous studies have assessed the efficacy of ZBTB20, including pro‐tumour cell growth, pro‐proliferation,^12^ immune‐regulating liver regeneration^11^ and liver metabolism.^22^ The cardioprotective effects of ZBTB20 were reported in our study for the first time. Several important new findings also came from the present study: (a) after ZBTB20 overexpressing for four weeks post‐MI, survival rate was increased and cardiac dysfunction was ameliorated, accompanied by decreased LV dilatation, cardiac hypertrophy, apoptosis, inflammation, oxidative stress and interstitial fibrosis; (b) ZBTB20‐overexpressed cardiomyocytes exposed to hypoxia showed decreased cell apoptosis; (c) ZBTB20 suppressed TNFα/ASK1/JNK1/2 axis; (d) treatment with a JNK1/2 agonist abolished anti‐apoptotic effect of ZBTB20 in vitro. Thus, ZBTB20 exerts protective effect against adverse cardiac remodelling post‐MI.

The mechanism of ZBTB20 on cardiac remodelling needs to be elucidated. Increased cardiomyocyte death causes acute loss of myocardial tissue, leading to structural and biomechanical changes.^21^ Thus, inhibiting apoptosis is promising to prevent LV remodelling after MI. The intrinsic apoptosis and extrinsic apoptosis pathways compose the whole apoptosis pathway.^23^ Intracellular stress activates the intrinsic apoptosis pathway which causes increased permeabilization of itochondrial outer membrane relying on Bax/Bak expression. Thus, mitochondria are promoted to release cytochrome c to cytosol, leading to the activation of caspase.^23^, ^24^ Extracellular stress signals activate extrinsic apoptosis pathway. TNFα is an initial trigger factor by binding to the death receptor, TNFα receptor 1 (TNFR1).^25^ Exceeded oxidative stress promotes the secretion of TNFα from resident mast cells and macrophages, which in turn injures myocardium.^26^ By binding to TNFR, TNFα also activates t‐Bid via the intrinsic mitochondrial apoptosis pathway, which involves ASK1 and JNK activation.^23^ Once activated, JNK increases the release of cytochrome c, leading to cell apoptosis.^23^ JNK1/2 deficiency in mice could resist to infarction‐refusion‐induced cardiomyocyte injury^9^ and hypertrophy.^27^ Oxidative stress also induces apoptosis via the ASK1/JNK pathway.^28^, ^29^, ^30^ ASK1 can be activated by various oxidants such as H_2_O_2_ and diamide.^31^ In resting cells, ASK1 binds with Trx, forming an inactive complex. Following stimulation, ASK1 dissociates from Trx and is activated by subsequent modifications.^32^ In our study, excessive oxidative stress occurred 4 weeks after MI, whereas ZBTB20 overexpression decreased ROS production and preserved the level of anti‐oxidants, leading to inactivation of ASK1. In addition, ZBTB20 also decreased inflammatory response post‐MI and reduced production and release of TNFα, leading to inactivation of ASK1/JNK pathway. ZBTB20 significantly inhibited ASK1/JNK activation in our MI and hypoxia model, whereas the JNK1/2 agonist almost completely counteracted the effect of ZBTB20 in vitro. Our in vivo data also confirmed that ASK1 overexpression blocked the protective effects of ZBTB20 on cardiac remodelling. Thus, our findings illustrate that ZBTB20 exerts its protective effect by inhibiting ROS‐TNFα/ASK1/JNK1/2 signalling.

In our in vitro study, a hypoxia model was used to mimic myocardial ischaemic stress during MI. However, after infarction, myocardial I/R injury is the most stress that heart tissue and cardiomyocytes suffer from.^33^ During I/R, the levels of NO•, O2 and NO3 are elevated.^34^ The increased ROS generation leads to decreased ATP production and augmented mitochondrial dysfunction, followed by cardiomyocyte death.^34^ Although I/R model was not adopted here, ZBTB20 also exerted anti‐oxidative effect in the hypoxia model, thus decreasing cell apoptosis. Further studies using an I/R model are suggested to explore anti‐remodelling effects of ZBTB20.

In summary, ZBTB20 protects against cardiac remodelling after MI through anti‐apoptotic, anti‐inflammatory, anti‐oxidative stress, antifibrotic and antihypertrophic effects. Thus, administration of ZBTB20 can be considered as a promising clinical strategy to treat post‐MI heart failure.

## CONFLICT OF INTEREST

The authors declare no conflict of interests.

## AUTHOR CONTRIBUTION

**Fangfang Li:** Data curation (equal); Writing‐original draft (equal); Writing‐review & editing (equal). **Yiming Yang:** Data curation (equal); Writing‐review & editing (equal). **Chuanyou Xue:** Data curation (equal). **Mengtong Tan:** Data curation (equal). **Lu Xu:** Formal analysis (equal); Writing‐original draft (equal). **Jianbo Gao:** Formal analysis (equal). **Luhong Xu:** Formal analysis (equal); Writing‐review & editing (equal). **Jing Zong:** Conceptualization (equal); Resources (equal). **Wenhao Qian:** Conceptualization (equal); Resources (equal).

## Supporting information

Fig S1

## Data Availability

All data generated or used during the study are available from the corresponding author by request.
