# Supplementary material for: Zinc Finger Protein ZBTB20 protects against cardiac remodelling post‐myocardial infarction via ROS‐TNFα/ASK1/JNK pathway regulation
Source: J Cell Mol Med. 2020 Oct 16;24(22):13383–96. doi: 10.1111/jcmm.15961 (PMC7701508; doi:10.1111/jcmm.15961)
Supplement: Supplementary file 1 — Fig S1 [file JCMM-24-13383-s001.docx]

**
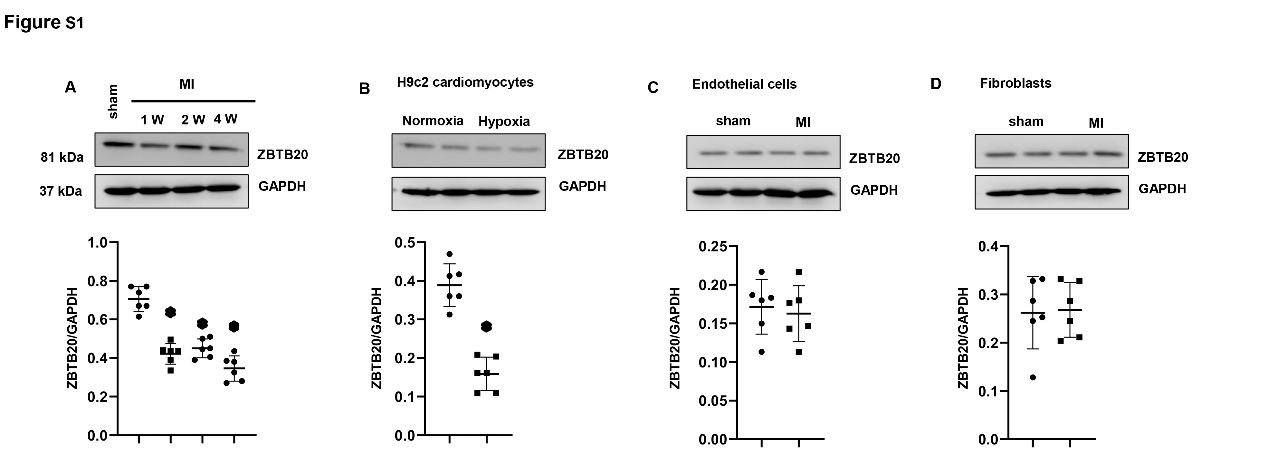
**

**Figure S1.** The expression level of ZBTB20 on heart tissue and different types of cells in heart

A. ZBTB20 protein level in mice heart after 1, 2 and 4 weeks of MI (n=6). B. ZBTB20 protein level in H9c2 cells exposed to hypoxia (n=6). C. ZBTB20 protein level in endothelial cells isolated from mice heart after four weeks of MI (n=6). D. ZBTB20 protein level in fibroblasts isolated from mice heart after four weeks of MI (n=6). *p<0.05 *vs.* sham/Normoxia. One-way analysis of variance followed by Tukey’s post hoc test was used in Figure S1A. Unpaired Student’s t-test was used in Figure S1B-D.
